# Lung Cancer Screening Communication in the US, 2022

**DOI:** 10.1001/jamanetworkopen.2024.42811

**Published:** 2024-11-04

**Authors:** Kalyani Sonawane, Ashvita Garg, Benjamin A. Toll, Ashish A. Deshmukh, Gerard A. Silvestri

**Affiliations:** 1 Department of Public Health Sciences, College of Medicine, Medical University of South Carolina, Charleston; 2 Medical University of South Carolina Hollings Cancer Center, Charleston; 3 Department of Medicine, Medical University of South Carolina Charleston

## Abstract

This cross-sectional study examines lung cancer screening communication between US clinicians and patients by smoking status and demographic, socioeconomic, and clinical characteristics.

## Introduction

Lung cancer is the leading cause of cancer death in the US.^1^ Through routine screening with low-dose computed tomography (LDCT), LC mortality can be averted.^2^ Current guidelines recommend lung cancer screening (LCS) for 50- to 80-year-old high-risk individuals (ie, former or current smoking), approximating 13 million LCS-eligible US individuals.^3,4^ However, in 2022, only 4.5% of LCS-eligible individuals were up to date according to the American Lung Association (ALA).^5^ Discussion about LCS with eligible individuals is foundational for LCS uptake. However, LCS communication by health care practitioners with high-risk adults remains unclear.^6^ The objective of this study was to examine LCS communication for US individuals at high risk by smoking status and demographic, socioeconomic, and clinical characteristics.

## Methods

This cross-sectional study analyzed data for participants aged 50 to 80 years from the 2022 Health Information National Trends Survey (HINTS)–6, a nationally representative survey of the civilian noninstitutionalized population conducted by the US National Cancer Institute. The HINTS-6 was administered using a mixed-mode (web- and paper-based) method; the overall response rate was 28.15%. This study was deemed exempt by the institutional review board at The Medical University of South Carolina because the data are deidentified and publicly available, in accordance with 45 CFR §46. Smoking status, sociodemographics, and clinical information are self-reported by participants. Participants with information on smoking status and who had at least 1 primary health care visit in the past year were identified. Participants were asked, “At any time in the past year, did a doctor or other health professional talk with you about having LDCT scan to check for lung cancer?” Participant responses “I have never heard of this test” and “No” were analyzed separately by smoking status. Combined estimates (never heard of LCS or not discussed LCS with a clinician) were also examined by smoking status. We followed the STROBE reporting guidelines. See the eMethods in Supplement 1 for statistical analysis.

## Results

A total of 929 participants (estimated population size, 29.0 million) who formerly smoked and 350 participants (11.9 million) who currently smoke were identified. Among those who formerly smoked, 18.1% (95% CI, 14.8%-21.5%) had never heard of LCS and 75.1% (95% CI, 70.8%-79.4%) never discussed LCS with their clinician (Figure). In the currently smoking group, 13.5% (95% CI, 7.7% to 19.4%) had never heard of LCS and 71.1% (95% CI, 63.2%-78.9%) did not discuss it with their clinician. Collectively, more than 80% of participants in both groups, regardless of sex, race, ethnicity, educational attainment, household income, urbanicity vs rurality, health insurance status, and unmet social determinants, had neither heard of LCS nor discussed LCS with a clinician (Table). Over 60% of individuals with a history of cancer or a comorbid lung did not discuss LCS with their clinicians.

**Figure.**
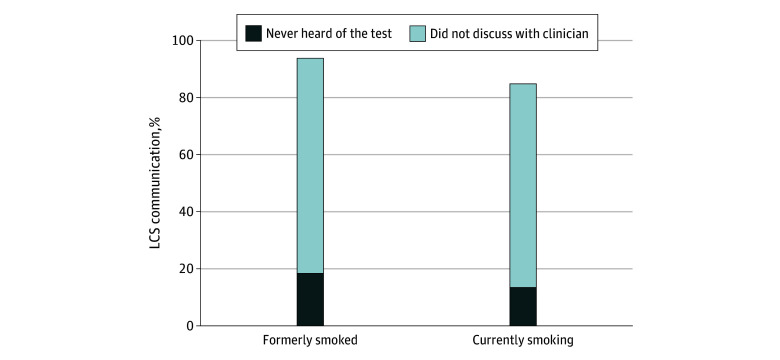
Lung Cancer Screening (LCS) Communication Among US Adults Aged 50 to 80 Years by Smoking Status The graph illustrates the weighted estimates of communication of LCS among individuals who formerly smoked and those who are currently smoking. Participants were asked, “At any time in the past year, did a doctor or other health professional talk with you about having a low-dose CT (LDCT) scan to check for lung cancer?” Information on smoking status was missing for 89 participants (1.5%), and information on primary care visit was missing for 71 participants (1.1%). Weighted numbers represent estimated population sizes derived from survey weights.

**Table.** Lung Cancer Screening Communication Among US Adults Aged 50 to 80 Years by Smoking Status and Sociodemographic and Clinical Characteristics^a^

Characteristic	Formerly smoked				Currently smoking			
	Participants, No. (%) [95% CI]			*P* value^b^	Participants, No. (%) [95% CI]			*P* value^b^
	Never heard of the test	Did not discuss with a clinician	Never heard or not discussed LCS		Never heard of the test	Did not discuss with a clinician	Never heard or not discussed LCS	
Total	182 (18.1) [14.8-21.5]	671 (75.1) [70.8-79.4]	853 (93.2) [90.9-95.6]	NA	52 (13.5) [7.7-19.4]	220 (71.1) [63.2-78.9]	272 (84.5) [79.7-89.3]	NA
Sex								
Male	88 (17.7) [12.9-22.6]	281 (75.4) [69.9-80.9]	369 (93.2) [90.0-96.3]	.97	25 (18.2) [7.80-28.4]	88 (64.5) [51.6-77.4]	113 (82.5) [75.0-90.0]	.45
Female	92 (18.5) [13.6-23.4]	386 (74.7) [67.8-81.7]	478 (93.3) [89.6-96.9]		27 (8.6) [4.3-12.8]	130 (77.9) [69.1-86.7]	157 (86.5) [80.0-93.0]	
Self-reported race and ethnicity								
Hispanic	26 (29.8) [15.6-43.9]	55 (63.2) [48.4-78.1]	81 (93.0) [84.3-100.0]	.80	7 (40.3) [0.0-83.6]	20 (53.1) [13.8-92.3]	27 (93.4) [84.0-100.0]	.65
Non-Hispanic Black	22 (12.4) [4.9-19.9]	83 (83.2) [74.0-92.4]	105 (95.6) [91.1-100.0]		8 (9.3) [2.9-15.7]	50 (75.7) [62.4-89.0]	58 (84.2) [72.8-95.6]	
Non-Hispanic White	116 (17.2) [13.6-20.9]	473 (75.5) [70.5-80.6]	589 (92.8) [89.8-95.7]		21 (8.2) [3.7-12.6]	125 (77.0) [69.1-84.9]	146 (85.0) [78.8-91.4]	
Other (American Indian or Alaska Native, Asian, Pacific Islander, and multiple races)	6 (19.1) [0.0-39.7]	28 (74.3) [53.9-94.6]	34 (93.3) [86.8-99.8]		10 (57.4) [24.2-903.6]	4 (21.6) [0.0-46.3]	14 (79.0) [51.3-100.0]	
Education level								
High school or less	56 (23.33) [15.2-31.4]	163 (71.4) [62.9-79.8]	219 (94.7) [90.9-98.5]	.42	21 (20.0) [6.1-33.8]	78 (61.6) [47.3-75.9]	99 (81.6) [72.3-91.0]	.47
College degree or higher	124 (15.8) [12.2-19.5]	506 (76.8) [72.3-81.3]	630 (92.6) [89.6-95.6]		31 (10.0) [5.3-14.6]	141 (76.0) [68.6-83.3]	172 (86.0) [79.9-92.1]	
Annual household income, $								
≤49 999	94 (19.8) [14.5-25.1]	298 (74.3) [68.7-80.0]	392 (94.1) [91.8-96.5]	.52	40 (15.6) [8.9-22.4]	146 (69.9) [61.2-78.6]	185 (85.6) [80.0-91.1]	.71
≥50 000	88 (17.2) [12.7-21.6]	372 (75.5) [69.3-81.8]	460 (92.7) [89.2-96.2]		12 (11.0) [0.8-21.3]	74 (72.3) [59.6-85.0]	86 (83.3) [74.0-92.7]	
Area of residence								
Urban	150 (17.8) [14.2-21.5]	566 (74.9) [70.0-79.7]	716 (92.7) [90.0-95.4]	.10	43 (13.1) [6.5-19.6]	175 (70.8) [61.8-79.8]	218 (83.9) [78.5-89.2]	.53
Rural	32 (20.0) [11.9-28.0]	105 (76.4) [68.2-84.5]	137 (96.3) [93.1-99.6]		9 (15.3) [3.5-27.1]	45 (72.5) [58.0-86.9]	54 (87.8) [77.0-98.5]	
Insurance status^c^								
Uninsured	6 (34.7) [4.0-65.4]	22 (61.3) [31.2-91.4]	28 (96.0) [90.5-100.0]	.42	3 (26.9) [0.0-66.2]	19 (71.8) [32.0-100]	22 (98.7) [96.0-100]	.01
Insured	173 (17.3) [14.2-20.5]	646 (75.8) [72.0-79.5]	819 (93.1) [90.6-95.6]		47 (11.8) [7.3-16.4]	201 (71.2) [63.9-78.5]	248 (83.0) [77.7-87.4]	
Comorbidity burden								
0	50 (18.4) [12.0-24.9]	180 (76.2) [66.7-85.8]	230 (94.7) [89.4-99.9]	.19	8 (6.0) [1.9-10.1]	65 (80.3) [68.0-92.6]	73 (86.2) [74.0-98.5]	.85
1	63 (16.7) [11.4-22.1]	241 (78.1) [72.2-84.0]	304 (94.8) [92.1-97.6]		15 (17.2) [2.9-31.4]	78 (67.5) [53.5-81.4]	93 (84.6) [76.5-92.8]	
≥2	69 (19.4) [13.6-25.2]	247 (70.7) [63.4-77.9]	316 (90.0) [85.5-94.6]		28 (17.0) [7.6-26.4]	75 (65.9) [55.6-76.3]	103 (82.7) [77.4-88.0]	
History of cancer								
Yes	29 (13.6) [8.0-19.1]	155 (75.5) [68.8-82.1]	184 (89.0) [83.6-94.5]	.12	9 (13.6) [1.4-25.8]	34 (61.3) [45.2-77.4]	43 (74.9) [62.5-87.4]	.11
No	152 (19.3) [15.4-23.2]	514 (75.0) [69.8-80.1]	666 (94.2) [91.4-97.1]		43 (13.6) [6.9-20.3]	185 (72.7) [63.3-81.9]	228 (86.2) [80.7-91.6]	
Lung condition (chronic lung disease, asthma, emphysema, or chronic bronchitis)								
Yes	23 (12.7) [5.6-19.9]	100 (71.0) [61.3-80.7]	123 (83.7) [75.9-91.6]	.01	23 (21.5) [10.7-32.3]	51 (62.4) [48.9-75.9]	74 (83.9) [76.0-91.8]	.11
No	159 (19.1) [15.2-23.0]	565 (75.7) [70.7-80.8]	724 (94.8) [92.3-97.4]		28 (10.6) [3.6-17.6]	165 (74.3) [64.7-83.8]	193 (84.8) [77.9-91.5]	
Unmet social determinants of health (transportation, housing, and food security), No.								
0	143 (17.6) [13.9-21.2]	548 (75.2) [70.3-80.1]	691 (92.8) [90.1-95.5]	.20	25 (8.9) [3.7-14.1]	137 (75.9) [66.8-85.1]	162 (84.9) [77.8-91.8]	.39
1-2	29 (23.4) [10.1-36.7]	87 (71.2) [57.2-85.3]	116 (94.6) [89.5-99.7]		20 (29.2) [9.3-49.0]	48 (49.8) [32.9-66.6]	68 (78.9) [67.7-90.2]	
3-4	9 (15.1) [0.6-29.7]	33 (81.8) [66.7-96.9]	42 (97.0) [93.9-100.0]		7 (12.6) [2.6-23.0]	35 (76.9) [63.0-91.0]	42 (89.5) [82.3-98.0]	

Abbreviations: LCS, lung cancer screening; NA, not applicable.

^a^ Participants were asked, “At any time in the past year, did a doctor or other health professional talk with you about having a low-dose CT (LDCT) scan to check for lung cancer?”

^b^ *P* values compare the combined outcome never heard or not discussed LCS.

^c^ The relative SE for uninsured strata was greater than 30%. Estimates may not be reliable and should be interpreted with caution.

## Discussion

This nationally representative cross-sectional study revealed that less than 16% of high-risk individuals had heard of LCS or discussed LCS with a health care practitioner. We also found that lack of LCS communication is pervasive across all sociodemographic and clinical subgroups. These findings are troubling because 13.1 million individuals meet the LCS eligibility criteria (ie, 20 pack-years and <15 years since quitting).^4^ Our data emphasize the need for increasing LCS communication in the US, specifically, increasing education and outreach to eligible individuals who can benefit from LCS. Limitations of HINTS include the cross-sectional nature, which precludes making causal inferences and the lack of availability of pack-years and years since quitting information. Nevertheless, our findings are consistent with a recent ALA survey that reported gaps in clinician LCS communication (73% never discussed their risk with a doctor) and LCS awareness (62% not familiar with the test) in the US.^6^ Implementing informational interventions at the clinic or community level could help improve LCS awareness and facilitate shared decision-making between eligible patients and health care practitioners.

## Notes

Supplement 1. **eMethods.** Statistical Analysis

Supplement 2.
Data Sharing Statement
