# Supplementary material for: Lung Cancer Screening Communication in the US, 2022
Source: JAMA Netw Open. 2024 Nov 4;7(11):e2442811. doi: 10.1001/jamanetworkopen.2024.42811 (PMC11536220; doi:10.1001/jamanetworkopen.2024.42811)
Supplement: Supplement 1. — eMethods. Statistical Analysis [file jamanetwopen-e2442811-s001.pdf]

Supplemental Online Content

Sonawane K, Garg A, Toll BA, Deshmukh AA, Silvestri GA. Lung cancer screening communication in the US, 2022. *JAMA Netw. Open*. 2024;7(11):e2442811. doi:10.1001/jamanetworkopen.2024.42811

eMethods. Statistical Analysis

This supplemental material has been provided by the authors to give readers additional information about their work.

© 2024 Sonawane K et al. *JAMA Network Open.*

eMethods. Statistical Analysis

A survey design–adjusted Wald F test was used. Statistical significance was tested at 2-sided $P < .05$. All analyses were conducted in January 2024 and performed with SAS statistical software version 9.4 (SAS Institute) using PROC SAS SURVEY procedures, which included weight, cluster, and strata statements to adjust for the complex survey design.

© 2024 Sonawane K et al. *JAMA Network Open.*
