# Supplementary material for: Lung Cancer Screening Communication in the US, 2022
Source: JAMA Netw Open. 2024 Nov 4;7(11):e2442811. doi: 10.1001/jamanetworkopen.2024.42811 (PMC11536220; doi:10.1001/jamanetworkopen.2024.42811)
Supplement: Supplement 2. — Data Sharing Statement [file jamanetwopen-e2442811-s002.pdf]

Data Sharing Statement

Sonawane. Lung Cancer Screening Communication in the US, 2022. *JAMA Netw Open*. Published November 04, 2024. doi:10.1001/jamanetworkopen.2024.42811

Data
Data available: Yes
Data types: Deidentified participant data
How to access data: https://hints.cancer.gov/
When available: With publication

Supporting Documents
Document types: None

Additional Information
Who can access the data: Anyone
Types of analyses: Per HINTS guidelines
Mechanisms of data availability: Per HINTS policies
